# Supplementary material for: Evidence for natural selection shaping the evolution of collective behavior among global Caenorhabditis elegans populations
Source: bioRxiv. 2026 Feb 5:2025.04.29.651269. Preprint. [Version 2] doi: 10.1101/2025.04.29.651269 (PMC12889462; doi:10.1101/2025.04.29.651269)
Supplement: Supplement 1 [file media-1.pdf]

Supplementary Information

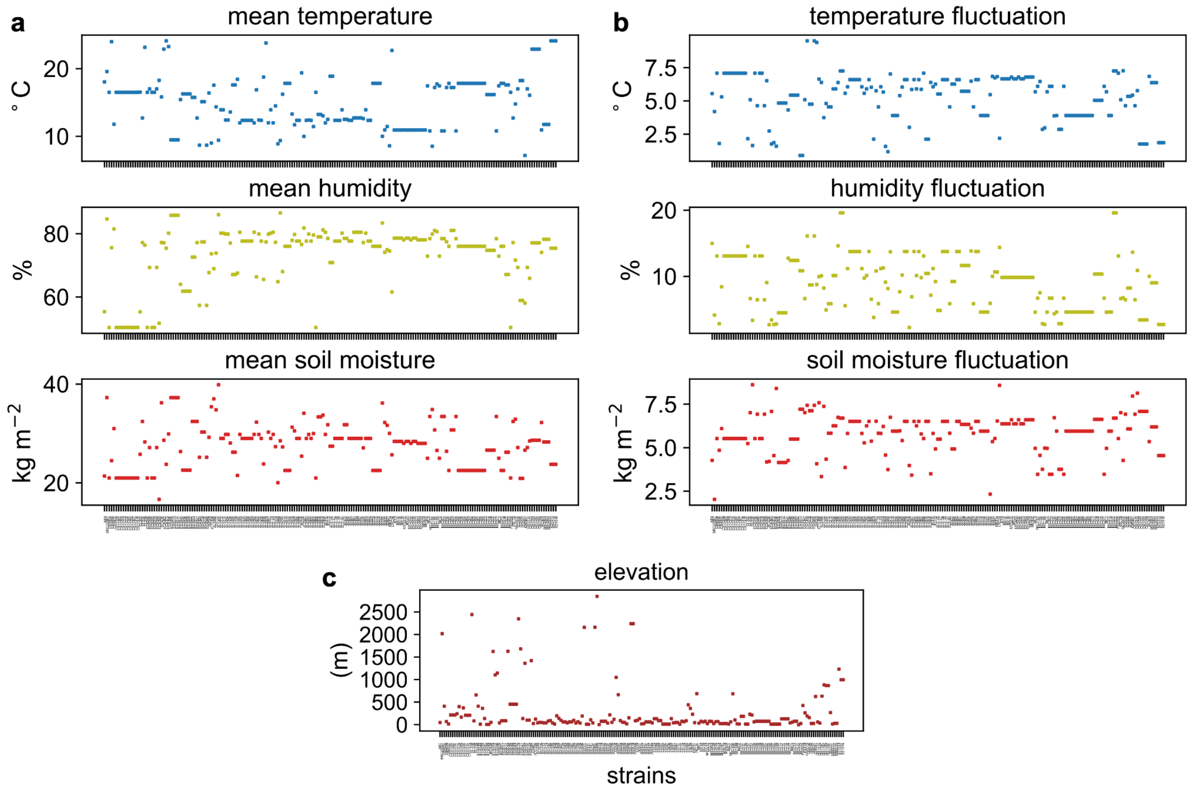

[Fig S1] Environmental information of the strain isolation sites. a. Near-surface temperature, near-surface relative humidity and upper column soil moisture of the isolation sites of the 190 wild strains, time-averaged over the period 2000-2014. b. Standard deviation of the temperature, relative humidity and soil moisture of the isolation sites of the wild strains, over the period 2000-2014 . c. Elevation of the isolation sites of the wild strains.

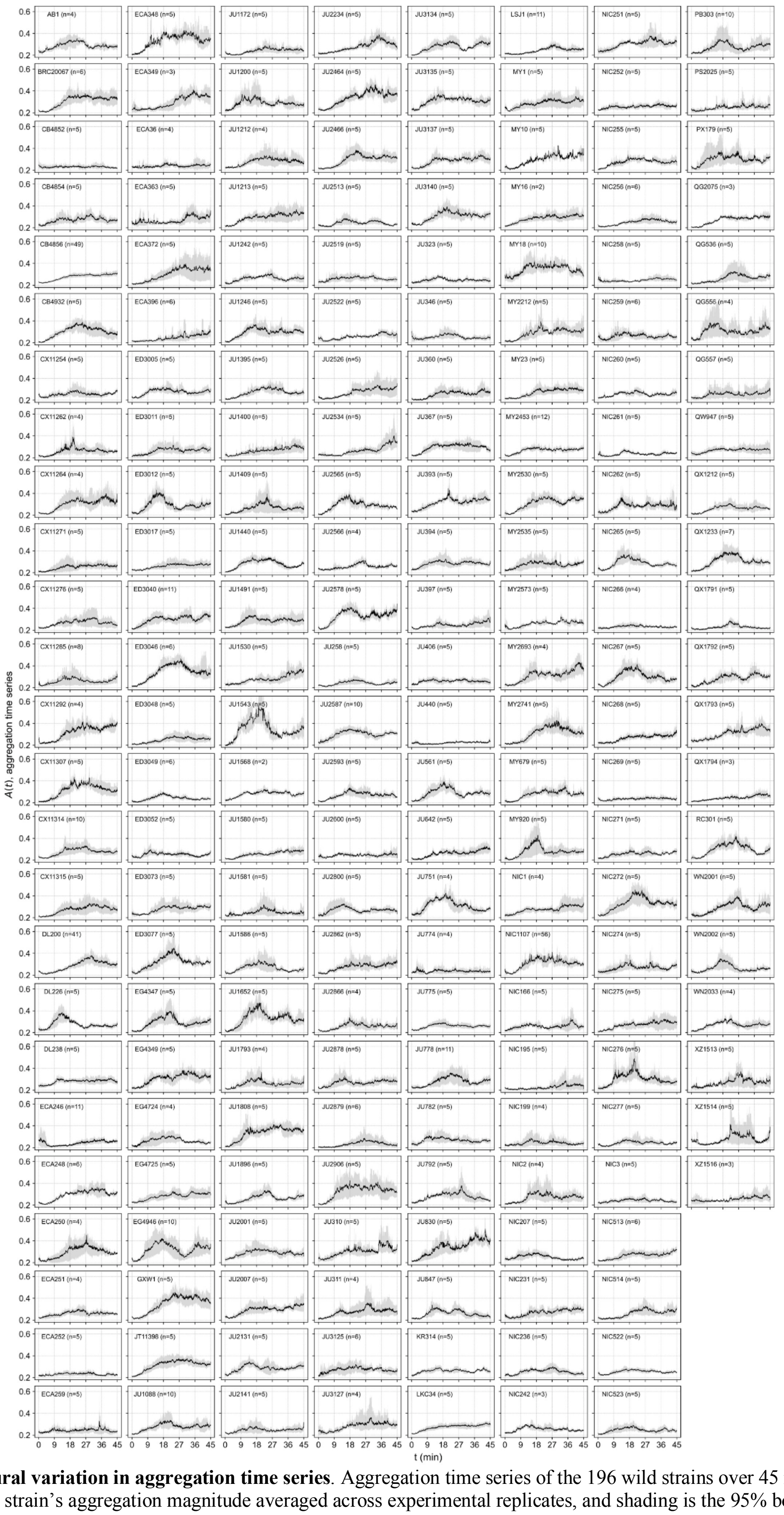

[Fig S2] Natural variation in aggregation time series. Aggregation time series of the 196 wild strains over 45 minutes. Line depicts a strain's aggregation magnitude averaged across experimental replicates, and shading is the 95% bootstrapped confidence interval to capture experimental variation.

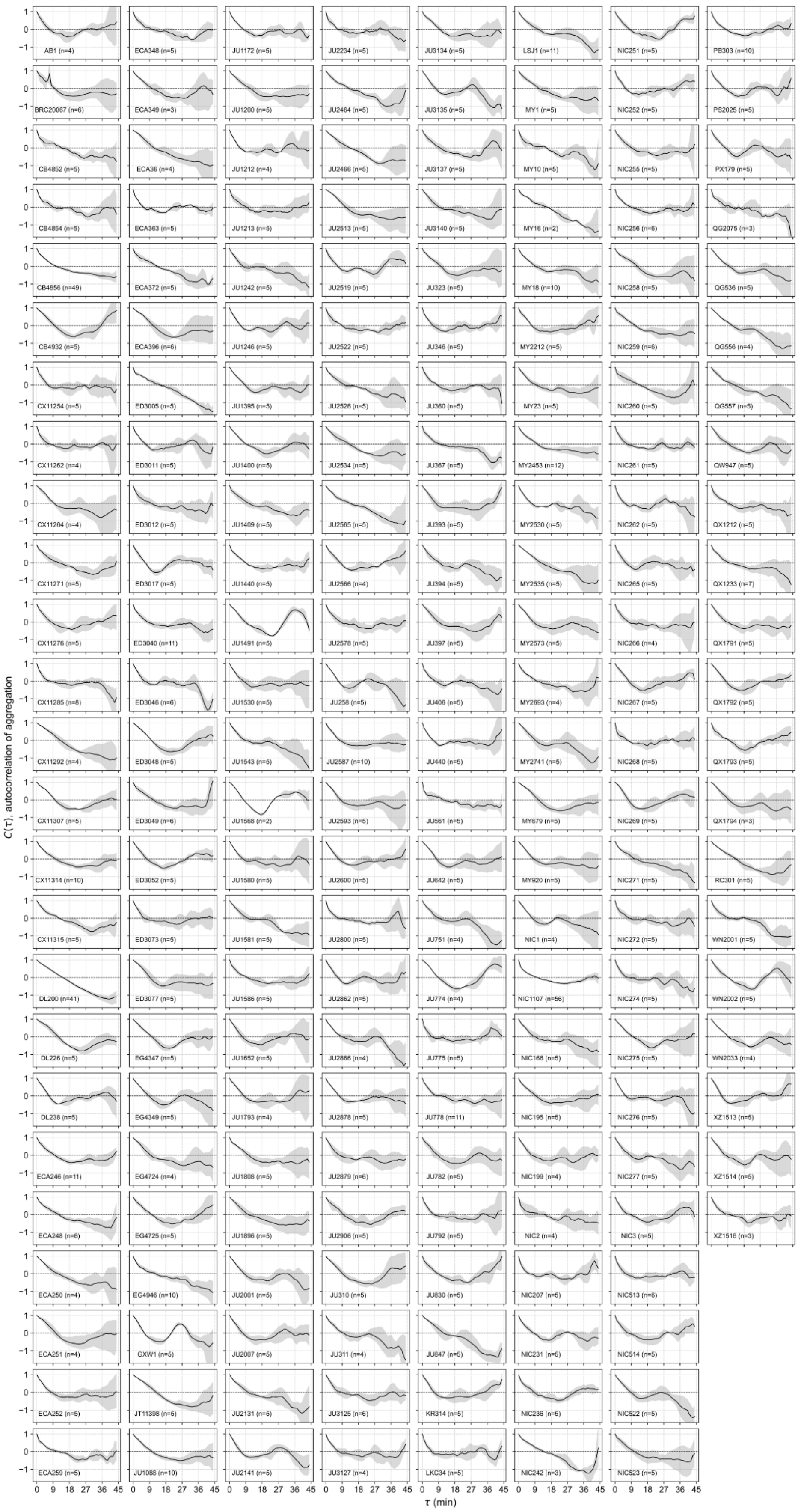

[Fig S3] Natural variation in aggregation dynamics. Autocorrelation function of the aggregation time series of the 196 wild strains as the measure of aggregation dynamics. Line depicts a strain's autocorrelation function averaged across experimental replicates, and shading is the 95% bootstrapped confidence interval to capture experimental variation.

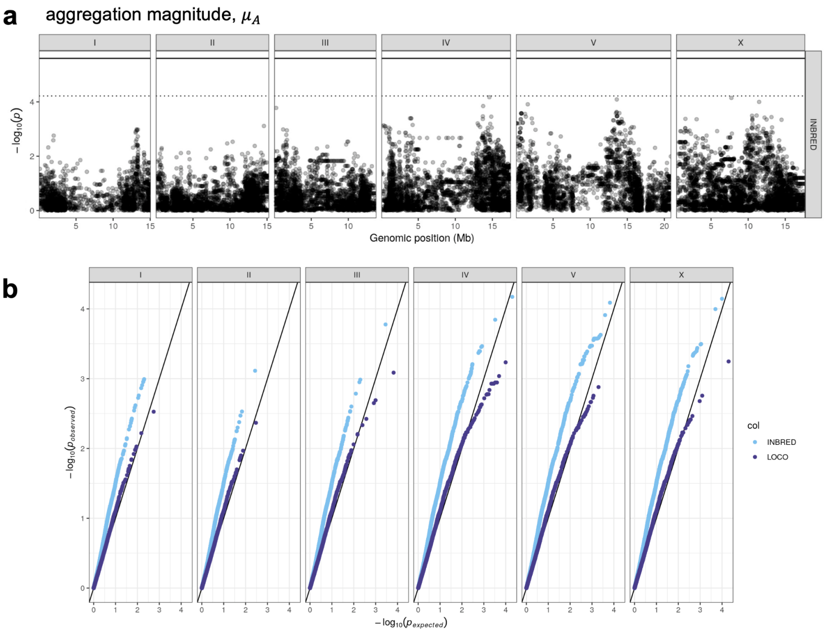

[Fig S4] GWAS result of the aggregation magnitude. a. Manhattan plot of aggregation magnitude showing genetic variants associated with natural variation in the phenotype (INBRED mapping). There is no genetic variant that surpassed the significant threshold. Dotted line refers to the EIGEN threshold while bold line refers to the Bonferroni score. b. Q-Q plot depicting the Genomic inflation factor of the INBRED and LOCO mapping. Solid line represents when the p-value obtained from the analysis matches the theoretical χ^2 p-values. Genomic inflation factor was 1.8031051 for the INBRED mapping and 1.1304331 for the LOCO mapping.

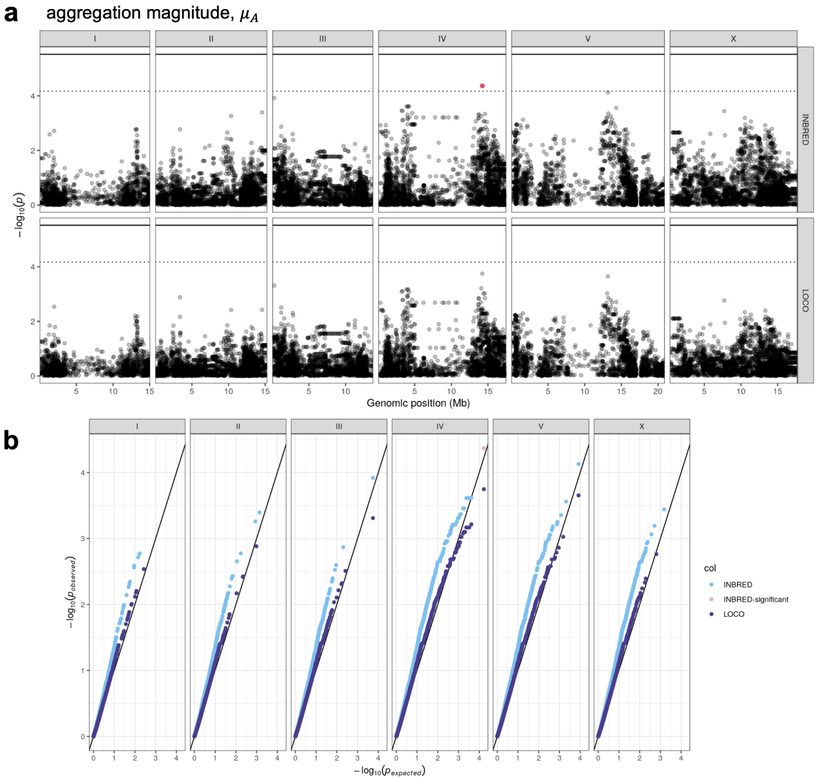

[Fig S5] GWAS result of the aggregation magnitude after pruning for population stratification using smartPCA. a. Manhattan plot of aggregation magnitude after pruning the outlier strains from smartPCA. Top panel refers to the result of the EIGEN algorithm and bottom panel the LOCO algorithm. Red dot refers to the peak marker of the genetic variant that surpassed the significant threshold of EIGEN score. b. Q-Q plot depicting the Genomic inflation factor of 1.4321477 for the INBRED mapping and 1.0482342 for the LOCO mapping.

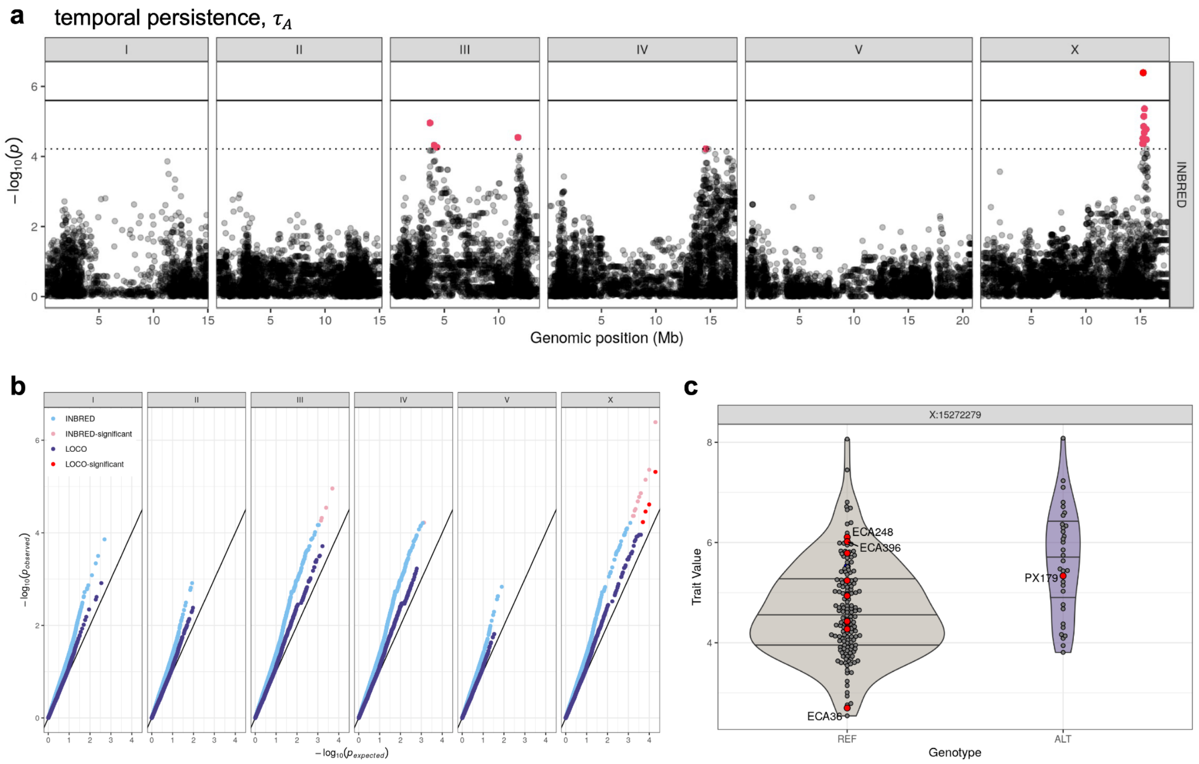

[Fig S6] GWAS result of the aggregation temporal persistence. a. Manhattan plot of aggregation temporal persistence showing the genetic variants associated with natural variation in the phenotype (INBRED mapping). Red dots refer to the peak markers of the genetic variants that surpassed the significant threshold of EIGEN score. Dotted line refers to the EIGEN threshold while bold line refers to the Bonferroni score. b. Q-Q plot depicting the Genomic inflation factors of the INBRED and LOCO mapping. Solid line represents when the p-value obtained from the analysis matches the theoretical χ^2 p-values. Genomic inflation factor was 1.6994635 for the INBRED mapping and 1.1259024 for the LOCO mapping. c. Temporal persistence trait split into two groups based on the genotypes at the peak marker on chromosome X. Some strains with high genetic divergence as defined by CaeNDR are annotated as red points. No outlier strains driving the divergence are observed, suggesting that the associations found are not driven by a few outlier strains.

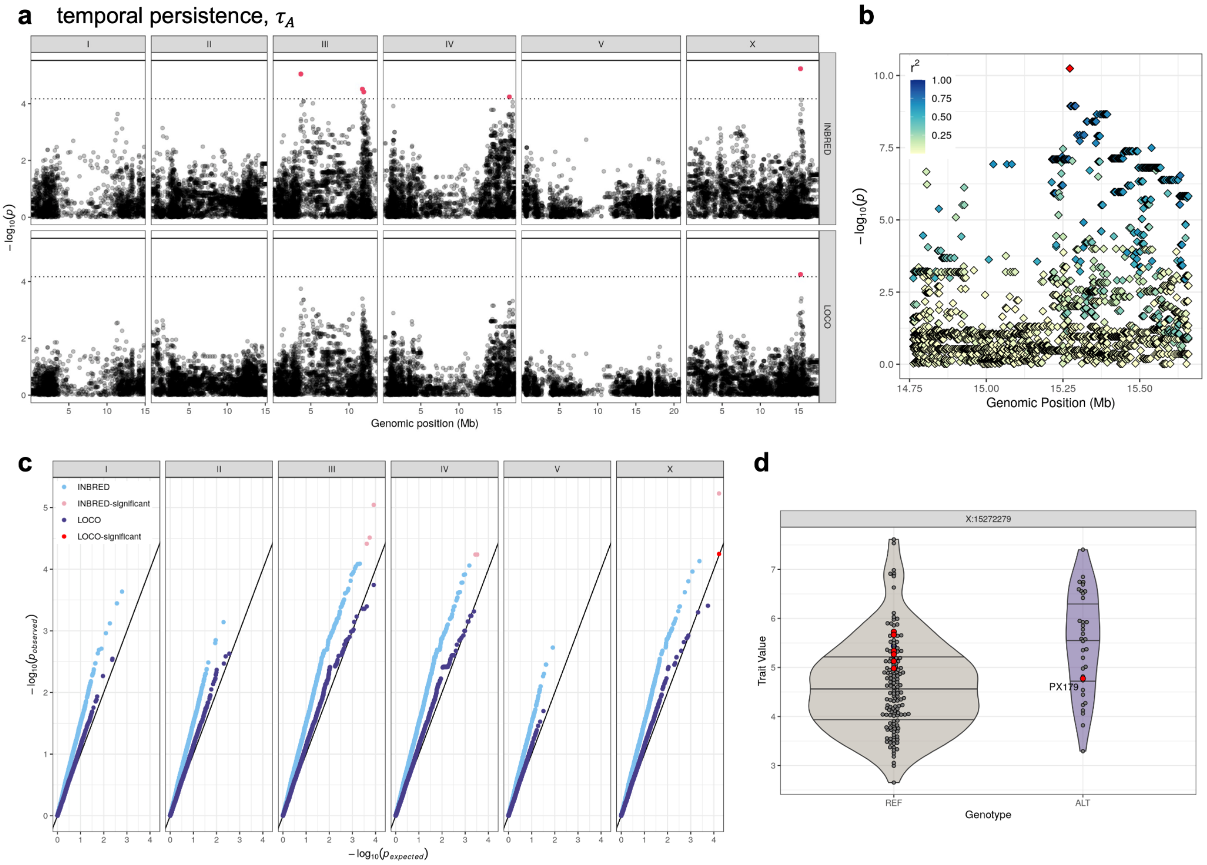

[Fig S7] GWAS result of the aggregation temporal persistence after pruning for population stratification using smartPCA. a. Manhattan plot of aggregation temporal persistence showing the genetic variants associated with natural variation in the phenotype. Top panel refers to the result of the EIGEN algorithm and the bottom panel the LOCO algorithm. Red dots refer to the peak markers of the genetic variants that surpassed the significant threshold of EIGEN score. Dotted line refers to the EIGEN threshold while bold line refers to the Bonferroni score. One QTL on chromosome X reports significant association in both the INBRED and LOCO mapping. c. Fine mapping result showing the SNP resolution mapping within the QTL at chromosome X. r^2 denotes the level of linkage to the red peak marker. c. Q-Q plot depicting the Genomic inflation factors of the INBRED and LOCO mapping. Solid line represents when the p-value obtained from the analysis matches the theoretical χ^2 p-values. Genomic inflation factor was 1.9977412 for the INBRED mapping and 1.1747531 for the LOCO mapping. d. Temporal persistence trait split into two groups based on the genotypes at the peak marker on chromosome X. Some strains with high genetic divergence as defined by CaeNDR are annotated as red points. No outlier strains driving the divergence are observed, suggesting that the associations found are not driven by a few outlier strains.

[Table S1] Source information of the CMIP6 climate model

source ID	institution ID	citation
CNRM-ESM2-1	CNRM-CERFACS	Seferian, Roland (2018). CNRM-CERFACS CNRM-ESM2-1 model output prepared for CMIP6 CMIP historical. Version 20241215. Earth System Grid Federation. https://doi.org/10.22033/ESGF/CMIP6.4068

[Table S2] Phylogenetic GLS aggregation magnitude model result

	Value	Std.Error	t-value	p-value
(Intercept)	0.0056946	0.08070428	0.0705615	0.9438
$H_{elevation}$	0.0606059	0.02573341	2.3551435	0.0196
μ_{temp}	-0.0956418	0.13703497	-0.697937	0.4861
μ_{humid}	-0.0139916	0.08374576	-0.167072	0.8675
μ_{moist}	0.0464189	0.13734817	0.3379652	0.7358
σ_{temp}	0.346845	0.18223536	1.9032805	0.0586
σ_{humid}	-0.1948629	0.19062997	-1.0222048	0.308
σ_{moist}	0.0643088	0.0558555	1.1513428	0.2511

[Table S3] Model selection on phylogenetic GLS aggregation magnitude

model	AIC
$\mu_A \sim \sigma_{temp} + H_{elevation}$	412.9324
$\mu_A \sim \mu_{temp} + \sigma_{temp} + H_{elevation}$	413.2035
$\mu_A \sim \mu_{moist} + \sigma_{temp} + H_{elevation}$	413.8245
$\mu_A \sim \mu_{humid} + \sigma_{temp} + H_{elevation}$	414.1748
$\mu_A \sim \sigma_{temp} + \sigma_{moist} + H_{elevation}$	414.4732
$\mu_A \sim \mu_{temp} + \sigma_{temp} + \sigma_{moist} + H_{elevation}$	414.6676

[Table S4] Phylogenetic GLS temporal persistence model result

	Value	Std.Error	t-value	p-value
(Intercept)	0.0291098	0.0768041	0.379014	0.7051
$H_{elevation}$	0.09216283	0.02448979	3.763316	0.0002
μ_{temp}	0.06999761	0.13041251	0.53674	0.5921
μ_{humid}	-0.0350763	0.07969859	-0.440112	0.6604
μ_{moist}	-0.0172041	0.13071057	-0.13162	0.8954
σ_{temp}	0.29121683	0.17342851	1.679175	0.0948
σ_{humid}	-0.2785657	0.18141743	-1.535496	0.1264
σ_{moist}	0.06991003	0.05315619	1.315181	0.1901

[Table S5] Model selection on phylogenetic GLS temporal persistence

model	AIC
$\tau_A \sim \mu_{humid} + H_{elevation}$	393.0655
$\tau_A \sim H_{elevation}$	393.2248
$\tau_A \sim \sigma_{temp} + H_{elevation}$	393.6564
$\tau_A \sim \mu_{moist} + H_{elevation}$	394.269
$\tau_A \sim \mu_{temp} + \sigma_{temp} + H_{elevation}$	394.4203
$\tau_A \sim \mu_{humid} + \sigma_{temp} + H_{elevation}$	394.5468

[Table S6] Covariance among GLS model predictors

	μ_{temp}	μ_{humid}	μ_{moist}	σ_{temp}	σ_{humid}	σ_{moist}	$H_{elevation}$
μ_{temp}	1	-0.2793096	-0.5081034	-0.5486109	-0.3747677	0.03813078	0.143063
μ_{humid}	-0.2793096	1	0.65387556	-0.306686	-0.450045	-0.0463122	-0.0963534
μ_{moist}	-0.5081034	0.65387555	1	-0.004855	-0.104441	-0.1541691	0.04301633
σ_{temp}	-0.5486109	-0.306686	-0.004855	1	0.65530001	0.00746113	-0.4870412

σ_{humid}	-0.3747677	-0.450045	-0.104441	0.65530001	1	0.31160359	-0.0145277
σ_{moist}	0.03813078	-0.0463122	-0.1541691	0.00746113	0.31160359	1	-0.0688574
$H_{elevation}$	0.143063	-0.0963534	0.04301633	-0.4870412	-0.0145277	-0.0688574	1

[Table S7] Geographic coordinates and elevation of the isolation sites of wild *C. elegans* strains

strain	latitude	longitude	elevation(m)
AB1	-34.93	138.59	48
BRC 20067	24.072954	121.169736	2018
CB4854	34.189	-118.131	409
CB4856	21.33	-157.86	69
CB4932	51.02	-3.10000000000002	15
CX11254	34.130138	-118.114835	214
CX11262	34.130138	-118.114835	214
CX11264	34.130138	-118.114835	214
CX11271	34.13712	-118.12532	241
CX11276	34.20111	-118.21198	399
CX11285	34.14331	-118.05496	166
CX11292	34.13531	-118.30582	372
CX11307	34.12946	-118.10987	208
CX11314	34.12946	-118.10987	208
CX11315	34.12946	-118.10987	208
DL200	9.03	38.74	2442
DL226	44.5633	-123.2821	83
DL238	19.11	-155.81	659
ECA246	34.189	-118.131	409
ECA248	37.44	-122.14	10
ECA250	34.096	-117.719	362
ECA251	34.1	-118.1	136
ECA348	37.775334	-122.253889	4
ECA349	32.765758	-117.236741	5
ECA36	-36.893333	174.745529	51
ECA363	20.723611	-156.304444	1623
ECA372	22.121667	-159.664444	1102
ECA396	19.449831	-155.235493	1144
ED3005	55.94	-3.36000000000001	39
ED3011	55.92	-3.19	85

ED3012	55.92	-3.19	85
ED3017	55.92	-3.19	85
ED3040	-26.166667	28.016667	1629
ED3046	-33.366667	19.316667	454
ED3048	-33.366667	19.316667	454
ED3049	-33.366667	19.316667	454
ED3052	-33.366667	19.316667	454
ED3073	-1.083333	36.65	2346
ED3077	-1.316667	36.8	1681
EG4347	44.04789	-123.07108	136
EG4349	40.771467	-111.87316	1360
EG4724	41.6288	-8.3476	99
EG4725	41.6288	-8.3476	99
EG4946	40.72596	-111.82184	1420
GXW1	30.542889	114.419828	23
JT11398	47.763944	-122.275484	124
JU1088	34.7613	138.0149	40
JU1172	-36.87	-73.04	51
JU1200	55.577	-4.60000000000002	48
JU1212	48.71	-3.81	39
JU1213	48.71	-3.81	39
JU1242	49.1269	1.9595	81
JU1246	49.12618	1.96152	88
JU1395	47.2199	0.04619	38
JU1400	37.3845	-5.988	14
JU1409	37.468	-5.637	196
JU1440	41.41307	2.15231	142
JU1491	46.63	1.06	96
JU1530	48.7015	2.1725	62
JU1543	48.7015	2.1725	62
JU1568	48.8092	2.3862	40

JU1580	48.7015	2.1725	62
JU1581	48.7015	2.1725	62
JU1586	46.63	1.06	96
JU1652	-34.86	-56.19	35
JU1793	49.12604	1.95114	66
JU1808	48.81257	2.395372	34
JU1896	37.999722	23.749673	191
JU2001	-21.1	55.5	2158
JU2007	50.7608	-1.32413000000003	9
JU2131	48.0497	-4.70499999999998	6
JU2141	46.6293	1.0512	107
JU2234	47.227	-1.58300000000003	38
JU2464	-13.155	-72.525	2162
JU2466	-13.257	-72.266	2845
JU2513	38.26016	22.0736	2
JU2519	38.7175	-9.14859999999999	77
JU2522	38.7175	-9.14859999999999	77
JU2526	38.7175	-9.14859999999999	77
JU2534	49.357921	0.097087	34
JU2565	44.958394	1.066213	218
JU2566	48.83886	2.44599	52
JU2578	48.87613	2.4315	118
JU258	32.73	-16.89	1049
JU2587	45.026713	3.872016	664
JU2593	48.822556	2.222334	88
JU2600	49.1242	1.9496	54
JU2800	48.79	2.331	49
JU2862	52.1944	0.1264	17
JU2866	34.0751	-118.4409	152
JU2878	19.29732	-99.098769	2240
JU2879	19.29732	-99.098769	2240

JU2906	43.8023	11.2806	84
JU310	46.63	1.06	96
JU311	44.42	4.4	132
JU3125	38.1127	13.3722	17
JU3127	38.11196	13.37274	20
JU3134	48.7015	2.1725	62
JU3135	48.7015	2.1725	62
JU3137	48.7015	2.1725	62
JU3140	48.71	-3.81	39
JU323	44.42	4.4	132
JU346	44.42	4.4	132
JU360	48.98	2.23	90
JU367	48.98	2.23	90
JU393	49.28	-0.319999999999993	13
JU394	49.28	-0.319999999999993	13
JU397	49.28	-0.319999999999993	13
JU406	49.28	-0.319999999999993	13
JU440	48.715	1.56	134
JU561	48.71	-3.81	39
JU642	48.84	2.5	48
JU751	48.84	2.5	48
JU774	38.683	-9.33999999999997	12
JU775	38.7175	-9.14859999999999	77
JU778	38.719	-9.14909999999998	65
JU782	38.7191	-9.15030000000002	85
JU792	43.06	0.24	441
JU830	48.52	9.05	362
JU847	48.46	7.461	230
KR314	49.28	-123.13	45
LKC34	-18	46	687
MY1	52.54	7.31	32

MY10	51.96	7.53	75
MY16	51.93	7.57	62
MY18	51.96	7.53	75
MY2212	54.346355	10.117737	22
MY23	51.96	7.53	75
MY2453	51.950777	7.536628	72
MY2530	54.346355	10.117737	22
MY2535	51.950723	7.599099	68
MY2573	51.950723	7.599099	68
MY2693	54.3491	10.11505	21
MY2741	54.3491	10.11505	21
MY679	54.346355	10.117737	22
MY920	54.346355	10.117737	22
NIC 1	43.279	5.3543	14
NIC 1107	43.716278	7.266935	63
NIC 166	46.72722	6.89775	684
NIC 195	38.545874	-28.37322	103
NIC 199	37.745428	-25.19929	18
NIC 2	43.279	5.3543	14
NIC 207	43.720505	7.24045	185
NIC 231	43.720505	7.24045	185
NIC 236	39.242006	-9.31341700000002	14
NIC 242	38.691765	-9.31608999999997	11
NIC 251	38.66621	-28.151364	230
NIC 252	38.661313	-28.147949	213
NIC 255	43.714834	7.266779	63
NIC 256	38.71829	-9.14875000000001	77
NIC 258	38.71829	-9.14875000000001	77
NIC 259	38.71829	-9.14875000000001	77
NIC 260	38.71829	-9.14875000000001	77
NIC 261	38.71829	-9.14875000000001	77

NIC 262	38.71829	-9.14875000000001	77
NIC 265	38.71829	-9.14875000000001	77
NIC 266	38.69237	-9.31592000000001	11
NIC 267	38.69237	-9.31592000000001	11
NIC 268	38.69237	-9.31592000000001	11
NIC 269	38.69237	-9.31592000000001	11
NIC 271	38.69237	-9.31592000000001	11
NIC 272	39.769459	-8.75635599999998	129
NIC 274	39.769459	-8.75635599999998	129
NIC 275	39.769459	-8.75635599999998	129
NIC 276	39.769459	-8.75635599999998	129
NIC 277	43.716441	7.266138	40
NIC 3	43.3636	5.3215	54
NIC 513	38.776038	-9.16713600000003	77
NIC 514	38.776038	-9.16713600000003	77
NIC 522	36.535679	-6.304441	0
NIC 523	36.535679	-6.29831000000001	24
PS2025	34.19	-118.13	425
PX179	44.035	-123.058	262
QG2075	33.951	-83.376	192
QG536	37.7679	-122.4415	157
QG556	34.421629	-119.702021	26
QG557	34.421629	-119.702021	26
QW947	-33.4213	-70.6106	625
QX1212	37.7502	-122.4331	59
QX1233	37.8804	-122.2838	34
QX1791	20.6344	-156.3935	631
QX1792	20.70554	-156.35475	882
QX1793	20.70559	-156.35678	866
QX1794	20.70559	-156.35678	866
RC301	47.99	7.84	268

WN2001	51.954422	6.303406	15
WN2002	51.975285	5.694834	29
WN2033	51.975	5.694	29
XZ1513	22.14787	-159.63105	1230
XZ1514	22.149	-159.668	995
XZ1516	22.149	-159.668	995

[Table S8] Comparison of AICc scores from analyzed MLPE models for pairwise genetic distances, with beta coefficients, *t*-statistics, and *p* values of fixed factors included in the top model.

Model	DF	AICc	β ± SE	*t*	*p* value
**Genetics ~ Geography**	**4**	**-117842.6**			
Geography			0.02 ± 0.00	38.76	< 0.001
Genetics ~ Environment	5	-117834.0			
Genetics ~ Geography + Environment	4	-116496.0			

[Table S9] Comparison of AICc scores from analysed MLPE models for pairwise phenotypic distances, with beta coefficients, *t*-statistics, and *p* values of fixed factors included in the top models.

Model	DF	AICc	β ± SE	*t*	*p* value
**Aggregation Magnitude ~ Genetics**	**4**	**35743.20**			
Genetics			2.12 ± 0.50	4.23	< 0.001
Aggregation Magnitude ~ Genetics + Elevation	5	35752.17			
Aggregation Magnitude ~ Genetics + Geography	5	35749.25			
Aggregation Magnitude ~ Genetics + Elevation + Geography	6	35758.41			
Aggregation Timescale ~ Genetics	4	39913.12			
**Aggregation Timescale ~ Genetics + Elevation**	**5**	**35532.22**			
Genetics			1.19 ± 0.50	2.38	0.017
Elevation			0.06 ± 0.01	6.91	< 0.001
Aggregation Timescale ~ Genetics + Geography	5	35571.36			
Aggregation Timescale ~ Genetics + Elevation + Geography	6	35537.78			
